# Virtual Surgical Planning of Deep Circumflex Iliac Artery Flap for Midface Reconstruction

**DOI:** 10.3389/fonc.2021.718146

**Published:** 2021-09-02

**Authors:** Yi-Fan Kang, Xiao-Ming Lv, Shi-Yu Qiu, Meng-Kun Ding, Shang Xie, Lei Zhang, Zhi-Gang Cai, Xiao-Feng Shan

**Affiliations:** ^1^Department of Oral and Maxillofacial Surgery, Peking University School and Hospital of Stomatology, Beijing, China; ^2^National Clinical Research Center for Oral Diseases, Beijing, China; ^3^National Engineering Laboratory for Digital and Material Technology of Stomatology, Beijing, China; ^4^Beijing Key Laboratory of Digital Stomatology, Beijing, China; ^5^Research Center of Engineering and Technology for Computerized Dentistry Ministry of Health, Beijing, China; ^6^National Medical Products Administration (NMPA) Key Laboratory for Dental Materials, Beijing, China

**Keywords:** virtual surgical planning, DCIA flap, midface reconstruction, dental implantation, maxillectomy, navigation system, surgical guide

## Abstract

**Objective:**

Midface reconstruction is challenging for functional and esthetic reasons. The present study analyzed the effect of virtual surgical planning (VSP) of the deep circumflex iliac artery (DCIA) flap for midface reconstruction.

**Patients and Methods:**

Thirty-four patients who underwent midface reconstruction with the DCIA flap were included in this retrospective study. Of the 34 patients, 16 underwent preoperative VSP, which used a three-dimensionally printed surgical guide, computer-assisted navigation system, and pre-bent titanium implants to transfer VSP into real-world surgery. The other 18 patients underwent traditional midface reconstruction. The following were compared between the two groups: bony contact rate in the buttress region (BCR), dental arch reconstruction rate (DAR), surgical approach, position of vascular anastomosis, and dental implantation rate. The independent-samples *t*-test and Fisher’s exact test were used for analysis. *P* < 0.05 was considered statistically significant.

**Results:**

In total, 12 males and 22 females were included in this study. All patients underwent midface reconstruction using the DCIA flap at the same institution. The median age of patients was 33 years (range: 16–68 years). The average BCR and DAR values in the VSP group were 59.4% ± 27.9% and 87.5% ± 18.9%, respectively, which were significantly higher compared with the non-VSP group (P = 0.049 and P = 0.004, respectively). The dental implantation rate in the VSP group (50.0%) was significantly higher compared with the non-VSP group (11.1%; P = 0.023). The intraoral approach for tumor ablation and vascular anastomosis was the most frequent choice in both groups. There was no significant difference between the two groups. All patients were satisfied with facial symmetry postoperatively.

**Conclusions:**

VSP could effectively augment the effect of midface reconstruction with the DCIA flap. Stronger bone contact in the buttress region and higher DAR provide more opportunity for dental implantation, which might be the best solution to improve masticatory function in patients with midface defects.

## Introduction

Midface reconstruction is challenging for functional and esthetic reasons (1–4). Many subregions form the midface, including the orbital floor, the zygomaticomaxillary complex, and the alveolar ridge. Many techniques for midface reconstruction were recommended by Brown in 2010 (5), including obturation, the soft tissue flap, and the hard tissue flap. The deep circumflex iliac artery (DCIA) bone flap, also known as the iliac crest flap, is the recommended method of reconstruction in the Brown II, III, and IV classifications (5).

The DCIA flap was first introduced by Urken in 1989 for oromandibular reconstruction (6). Brown used it for maxillary reconstruction in 1996 (7). The most obvious advantage of the DCIA flap is that it provides a reasonable bone height, not only to support the midface buttress, but also for dental implants. The disadvantages are equally obvious, including the short pedicle length and the difficulty in raising the flap. For now, use of vascularized bone flaps and dental implants to functionally reconstruct the midface is a growing trend. There are numerous studies on the fibular flap. However, our recent study showed that bone of the fibular flap for maxillary reconstruction is unlike mandibular reconstruction, since it is absorbed constantly over time (8). This makes the DCIA flap popular at our institution.

In the last decade, with the development of virtual surgical planning (VSP) for reconstruction of oral and maxillofacial defects, the accuracy and safety of midface reconstruction has been greatly improved, and it provides more possibilities for surgeons. At present, with the help of VSP and navigation systems, maxillary tumor resection can be performed through the intraoral approach in many cases. It solves the problem of facial scarring observed with the traditional Weber Ferguson approach. In addition, VSP achieves both esthetic and functional reconstruction results. However, there are few studies on midface reconstruction using the DCIA flap and VSP. The present study aimed to analyze the effect of VSP for midface reconstruction with the DCIA flap.

## Materials and Methods

### Patient Selection

Patients who underwent midface reconstruction with the DCIA flap at the Department of Oral and Maxillofacial Surgery, Peking University School and Hospital of Stomatology, Beijing, China, between May 2017 and December 2020 were enrolled in this retrospective study. The inclusion criteria were (1) a maxillary defect after tumor resection requiring reconstruction with the DCIA flap, (2) at least one iliac bone segment used to reconstruct the alveolar ridge, and (3) a normal occlusal relationship before surgery. The exclusion criteria was the flap did not survive. There were 34 patients who met the criteria. Patients were divided in two groups according to whether they underwent preoperative VSP. In the VSP group, VSP was transferred into real-world surgery using one or more techniques, including three-dimensionally printed surgical guides, navigation systems, and pre-bent titanium implants. Patients in the non-VSP group underwent reconstruction surgery using the traditional method. This study adhered to the principles of the Declaration of Helsinki in terms of medical protocols and ethics and was approved by the institutional ethics committee (PKUSSIRB - 202055065).

### Virtual Surgical Planning

**Figure 1:**
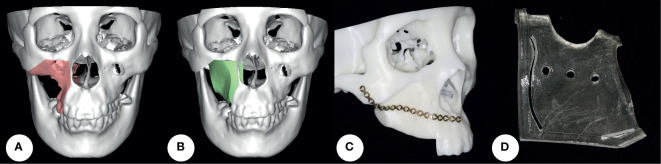
Virtual surgical planning. **(A, B)** Maxillectomy and reconstruction were simulated using software. **(C)** A resin stereo model was three-dimensionally printed to pre-bend the titanium plate. **(D)** A surgical guide was used for DCIA flap harvesting and shaping. DCIA flap: deep circumflex iliac artery bone flap.

In the VSP group, preoperative computed tomography (CT) scans (120 kV, 25 mAs, SW = 1.25 mm) of the head and neck region and the iliac region were performed for VSP. The aim of VSP was to precisely reconstruct the midface buttress and alveolus for later dental implantation based on the symmetry of the midface contour. Maxillectomy and reconstruction were simulated using ProPlan CMF 3.0 (Materialize, Belgium) and iPlan CMF 3.0 (BrainLab, Germany). With the concept of occlusion-driven reconstruction, the position of the iliac bone segment not only met the requirement for implantation, but also met the contour of the maxilla. A resin stereo model was three-dimensionally printed to pre-bend the titanium plate. A surgical guide was used for DCIA flap harvesting and shaping (**Figure 1**). Maxillectomy was performed under guidance of the navigation system. After DCIA flap fixation, the location of bone grafts was also confirmed by the navigation system. In both groups, the titanium mesh was the first choice for orbital floor reconstruction.

### Bone Contact Rate in the Buttress Region

Postoperative CT scans were performed to evaluate bone contact between iliac bone segments and the remaining native maxilla in the buttress region. The main vertical buttresses in the maxilla include the zygomaticomaxillary buttress, the nasomaxillary buttress, and the pterygomaxillary buttress. The buttress was considered reconstructed only if the gap between the iliac bone segment and the remaining native maxilla was less than 1 mm in CT images. BCR was defined as the percentage of reconstructed buttress of the total lost buttress.

### Dental Arch Reconstruction Rate

**Figure 2:**
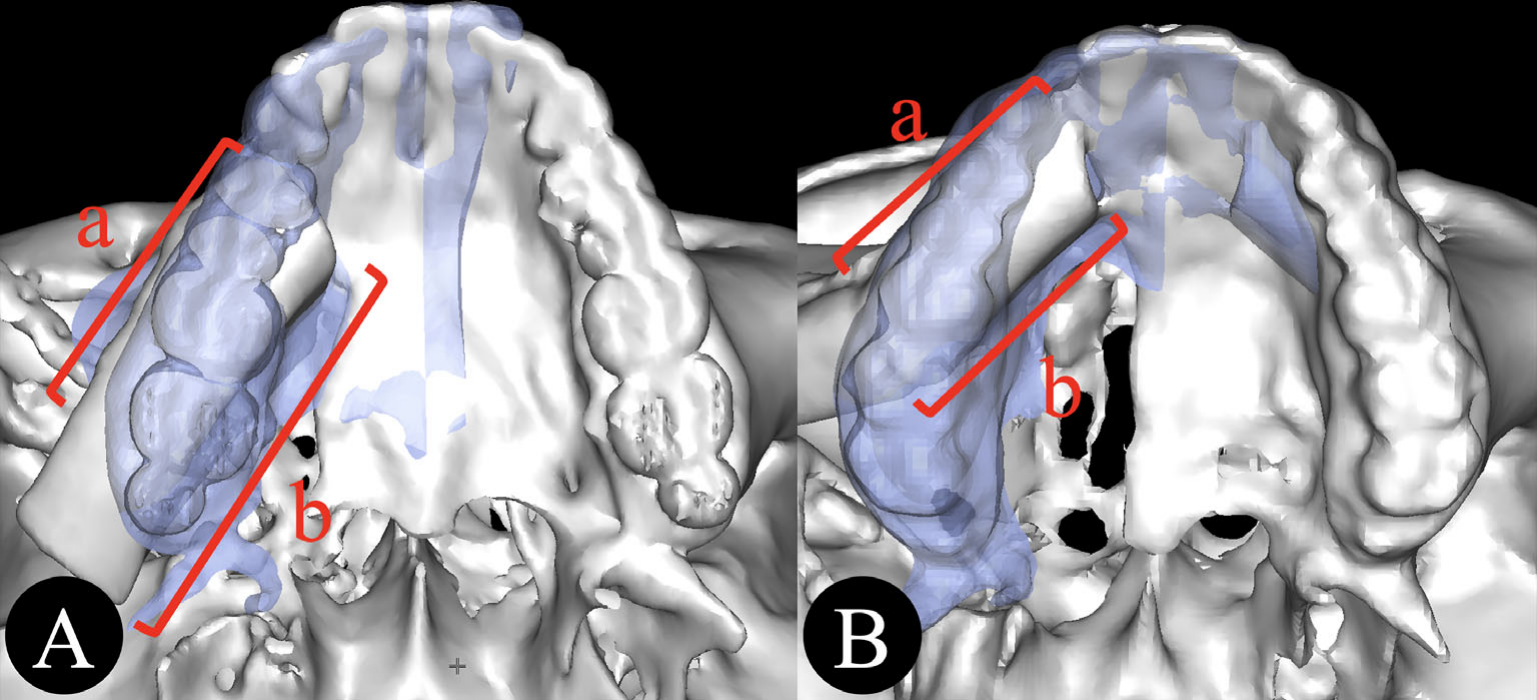
**(A, B)** Dental arch reconstruction rate. DAR = a/b (a, the length of the iliac bone overlapping the dental arch; b, the total length of iliac bone segments for alveolar reconstruction).

Postoperative CT scans were reconstructed in three dimensions using ProPlan CMF 3.0 (Materialize, Belgium). The unaffected side of the dental arch of the maxilla was mirrored as a reference. DAR was defined as the percentage of iliac bone length overlapping the mirrored dental arch for alveolar reconstruction (**Figure 2**). This variable could reflect the intermaxillary relationship.

### Other Variables

The following were also assessed and compared between groups: the surgical approach, the position of vascular anastomosis, and the dental implantation rate. The surgical approach included the intraoral approach and the Weber Ferguson approach. The position of vascular anastomosis included the intraoral recipient area and the neck recipient area.

### Statistical Analysis

All statistical analyses were performed using SPSS 20 (SPSS Inc., USA). The independent-samples *t-*test was used to investigate differences between the two groups in BCR and DAR. Fisher’s exact test was used to identify differences in dental implantation rate, surgical approach, and position of vascular anastomosis. A *P* value of <0.05 was considered statistically significant.

## Results

### Comparison of VSP and Non-VSP Groups

There were 34 patients (12 males and 22 females) enrolled in this study. All patients underwent midface reconstruction with the DCIA flap. VSP was used in 16 cases. The median age of patients was 33 years (range: 16–68 years). Patients’ characteristics and Brown defect classifications in the VSP and non-VSP groups are shown in **Table 1**. All patients were satisfied with postoperative facial symmetry.

**Table 1:** Patients’ characteristics.

Variable	Clinical details	
	VSP group	Non-VSP group
Number of patients	16	18
Sex		
Male	7	5
Female	9	13
Mean age (years, range)	33.8 (16–46)	33.4 (17–68)
Disease		
Benign tumor	13	16
Malignant tumor	3	2
Brown defect classification		
II	11	14
III	5	4
Segment of iliac bone		
One	11	12
Two	5	6

VSP, virtual surgical planning.

The easiest buttress to reconstruct was the nasomaxillary buttress, followed by the zygomaticomaxillary buttress and the pterygomaxillary buttress. The average BCR in the VSP and non-VSP groups was 59.4% ± 27.9% and 37.0% ± 35.5%, respectively. The average BCR in the VSP group was significantly higher compared with the non-VSP group (*P* = 0.049).

The average DAR in the VSP and non-VSP groups was 87.5% ± 18.9% and 64.9% ± 23.4%, respectively. The average DAR in the VSP group was significantly higher compared with the non-VSP group (*P* = 0.004).

The dental implantation rate in the VSP group was 50.0%, which was significantly higher compared with the non-VSP group (11.1%; *P* = 0.023). All patients were finished denture restoration in VSP group, seven of them were fixed denture and one was removable denture. Although two patients underwent dental implantation in none-VSP group, none of them finished denture restoration. The intraoral surgical approach and intraoral vascular recipient area were the first choice in the VSP group. The intraoral surgical approach was also popular in the non-VSP group, but the most frequent choice of vascular recipient area was the neck. There was no significant difference between the two groups (**Table 2**).

**Table 2:** Choice of surgical approach and vascular recipient area in the VSP and non-VSP groups.

Variables	Percentages		*P* value
	VSP group	Non-VSP group	
Intraoral surgical approach	75.0%	66.7%	0.715
Intraoral vascular anastomosis	62.5%	38.9%	0.303

VSP, virtual surgical planning.

### Case Presentation

**Figure 3:**
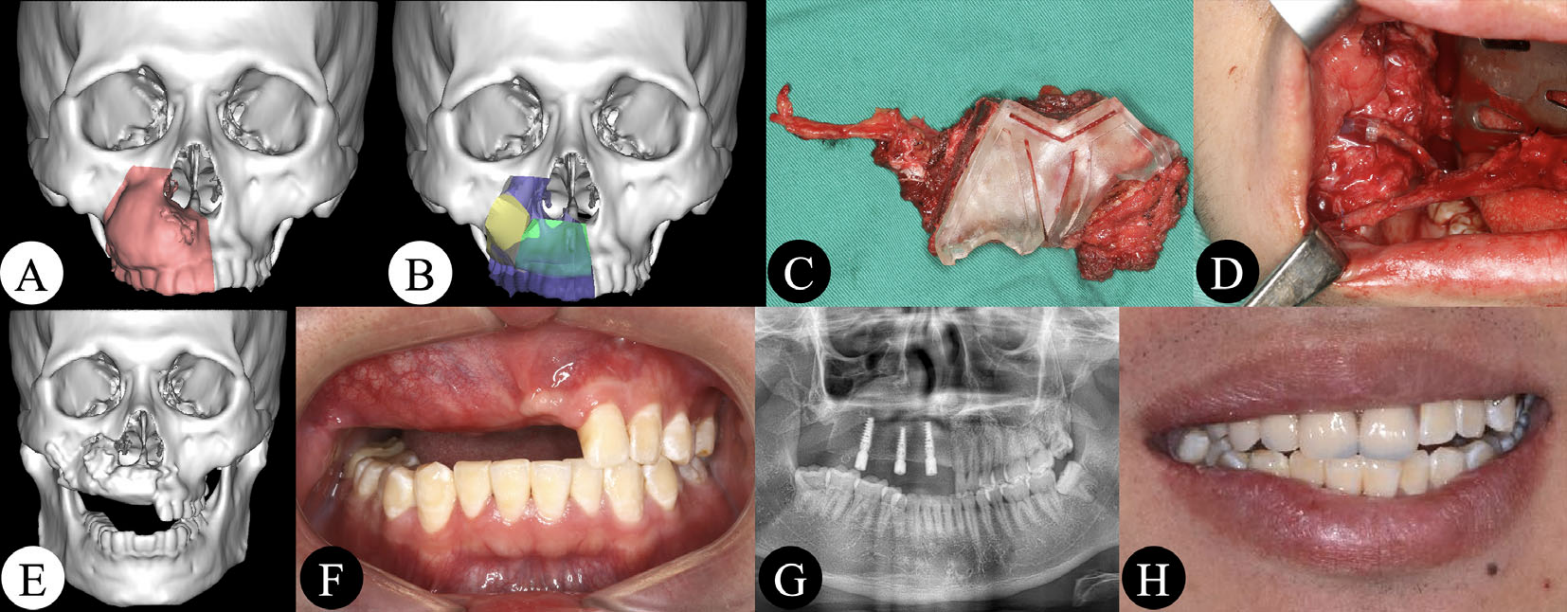
**(A, B)** Maxillectomy and reconstruction were simulated using software. **(C)** DCIA flap harvesting and shaping under guidance of a three-dimensionally printed surgical guide. **(D)** Intraoral vascular anastomosis. **(E, F)** Postoperative CT scan and intraoral picture showing good bone contact and a good intermaxillary relationship. **(G)** Three dental implants were inserted. **(H)** An implant-based removable denture was applied.

A 23-year-old male patient was treated for right maxillary ossifying fibroma. VSP was performed preoperatively. During surgery, tumor resection was performed under guidance of the navigation system through the intraoral approach. A three-dimensionally printed surgical guide was used to assist flap harvesting and shaping. Two segments of the DCIA flap were used to reconstruct the defect after maxillectomy. An intraoral vascular anastomosis was performed, and the flap was fixed. The myo-fascial flap of the external oblique abdominis was used to repair the soft tissue defect. Three dental implants were inserted into iliac bone at 6 months postoperatively. Finally, an implant-based removable denture was applied. The patient was satisfied with the functional and esthetic results (**Figure 3**).

## Discussion

Some excellent teams have reported their work on midface reconstruction with the DCIA flap (9–11). However, to our knowledge, the sample size of the present study is the largest to compare VSP with traditional surgery for midface reconstruction with the DCIA flap. Compared with studies using the fibular flap, the number of studies that used the DCIA flap for midface reconstruction is very small. This is likely because the DCIA flap has some obvious disadvantages. First, the pedicle of the DCIA flap is shorter than that of the fibular flap, especially when the recipient area is the neck. Bianchi et al. (9) and Baliarsing et al. (10) solved this problem using vein grafts. Intraoral anastomosis is another good option to effectively reduce the need for a sufficient pedicle length. It was introduced by Gaggl (12) and is performed at our institution on a regular basis (13). In this study, intraoral anastomosis was the first choice in both the VSP and non-VSP groups. The second disadvantage of the DCIA flap is the difficulty in raising the flap. Abdominal herniation can be avoided by carefully closing the wound; however, this complication did not occur in any patient in this study. Moreover, different surgical techniques and modifications appear to reduce complications, such as DCIA flaps with only an inner cortex (14) and a medial approach to harvest the DCIA flap (15). Thus, in our experience, the DCIA flap is the same as the fibular flap in terms of midface reconstruction, and it is better than the fibular flap when used for Brown class II and III defects.

The advantages of VSP have been well-documented in recent years. VSP helps surgeons to virtually visualize the tumor and to locate the resection margin. The length and angle of bone segments can be cut precisely using surgical guides. Using navigation systems, tumor resection and bone flap placement can be finished with less surgical exposure. VSP also enhances functional and esthetic outcomes (16–19). However, one disadvantage of VSP is that it cannot consider soft tissues, which play an important role in dental implantation and restoration (20). The DCIA flap is more bulky than the fibular flap, although some modifications have been made at our institution to reduce the volume of soft tissue (21). To our knowledge, dental implantation at the second stage might be suitable in most cases requiring the DCIA flap. The bulky soft tissue of the DCIA flap made the condition unfavorable for implants. In this study, all dental implants were inserted at the second stage. Surplus soft tissue of the DCIA flap was removed at the same time as dental implantation was performed. A gingiva graft was also essential for the long-term effect of dental implantation.

The BCR is a reliable indicator to evaluate the results of VSP (20, 22). The average BCR in the VSP group was significantly higher compared with the non-VSP group, which proved that patients benefited from VSP. The high BCR in the VSP group became one of the main reasons for a high dental implantation rate. Another reason for a high dental implantation rate was that VSP helped us to achieve a suitable intermaxillary relationship; a poor relationship might cause failure of dental implant-based restoration after reconstruction surgery (23). DAR reflected the degree of matching of iliac bone and the dental arch in a horizontal direction. The higher the DAR, the better the intermaxillary relationship.

Although the cost of VSP was not considered in this study, Mazzola et al. (24) demonstrated that a higher material cost might be encountered by patients undergoing VSP. Hua et al. (25) reviewed the literature and found that VSP can improve the dental implant rehabilitation rate after jaw reconstruction. Thus, the higher cost seems to be justified to achieve a better quality of life.

**Figure 4:**
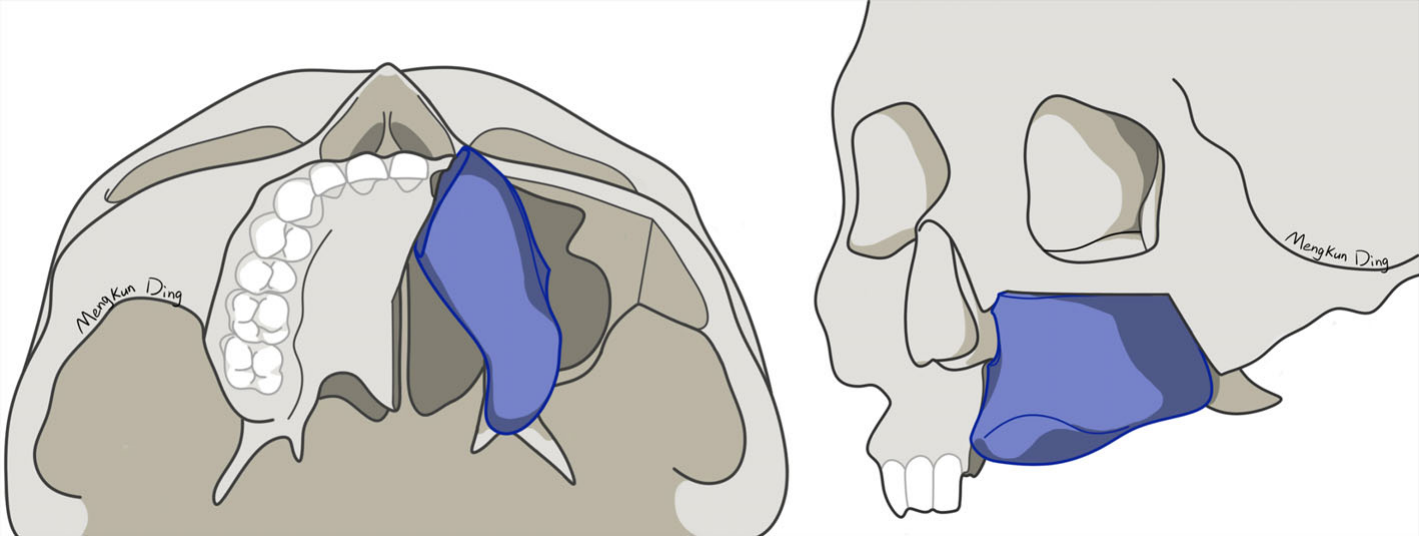
Shaping and reconstruction strategy for Brown class II defects.

**Figure 5:**
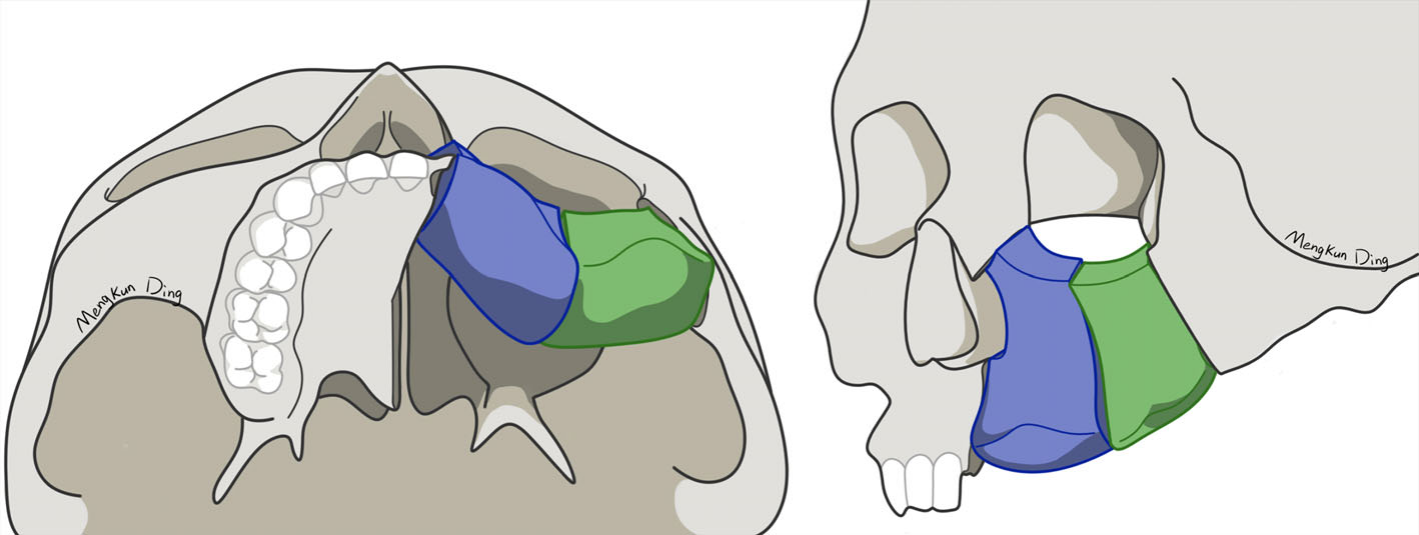
Shaping and reconstruction strategy for Brown class III defects.

Unlike the fibula, iliac bone has an irregular shape. It is difficult for surgeons to perform iliac bone shaping, depending on their own experience. VSP, surgical guides, and navigation systems can help surgeons to precisely shape and fix iliac bone, which means better bone contact between iliac bone and the maxilla. A higher bone contact rate would result in better bone union (22). However, there are few studies on how to shape and place the DCIA flap. In most previous studies, the DCIA flap was only shaped in one segment for midface reconstruction, using its natural curvature to mimic maxillary alveolar ridge (5, 9–11). However, we use different shaping and reconstruction strategies for Brown class II and III defects. For Brown class II defects, a one-segment DCIA flap is recommended to primarily reconstruct the nasomaxillary and pterygomaxillary buttresses (**Figure 4**). In our experience, direct contact between the iliac bone and the zygoma should be avoided in one-segment situations, because it will lead to buccal shift of iliac bone from the dental arch, resulting in an incorrect intermaxillary relationship. For Brown class III defects, a two-segment DCIA flap is recommended to reconstruct the nasomaxillary and zygomaticomaxillary buttresses (**Figure 5**). The orbital floor and alveolus could both be reconstructed using this method. Irrespective of the defect classification, we strongly suggest that the end of the iliac bone segment, which is used for alveolar reconstruction, should reach the position of the maxillary first molar as far as possible to achieve a functional result.

In conclusion, VSP could effectively augment the effect of midface reconstruction with the DCIA flap. Stronger bone contact in the buttress region and higher dental arch reconstruction rate provide more opportunity for dental implantation, which might be the foundation to improve masticatory function in patients with midface defects. However, the majority of this study were benign tumors, the results might not be directly applied to those malignant patients who need post-operative radiotherapy or chemotherapy. The new reconstruction strategy should be performed in more cases to prove its effectiveness and practicability.

## Data Availability Statement

The raw data supporting the conclusions of this article will be made available by the authors, without undue reservation.

## Ethics Statement

The studies involving human participants were reviewed and approved by Peking University School and Hospital of Stomatology Institutional Ethics Committee. Written informed consent to participate in this study was provided by the participants’ legal guardian/next of kin. Written informed consent was obtained from the individual(s) for the publication of any potentially identifiable images or data included in this article.

## Author Contributions

Y-FK: study design, manuscript preparation, data analysis and statistical analysis. X-ML, S-YQ, and M-KD: data collection and virtual surgical planning. Z-GV, X-FS, LZ, and SX: manuscript review and approved the final manuscript for submission. All authors contributed to the article and approved the submitted version.

## Funding

This work was supported by Capital’s Funds for Health Improvement and Research (2020-2-4102) and National Program for Multidisciplinary Cooperative Treatment on Major Diseases (PKUSSNMP-202015).

## Conflict of Interest

The authors declare that the research was conducted in the absence of any commercial or financial relationships that could be construed as a potential conflict of interest.

## Publisher’s Note

All claims expressed in this article are solely those of the authors and do not necessarily represent those of their affiliated organizations, or those of the publisher, the editors and the reviewers. Any product that may be evaluated in this article, or claim that may be made by its manufacturer, is not guaranteed or endorsed by the publisher.
